# Selective Killing of Activated T Cells by 5-Aminolevulinic Acid Mediated Photodynamic Effect: Potential Improvement of Extracorporeal Photopheresis

**DOI:** 10.3390/cancers12020377

**Published:** 2020-02-06

**Authors:** Sagar Darvekar, Petras Juzenas, Morten Oksvold, Andrius Kleinauskas, Toril Holien, Eidi Christensen, Trond Stokke, Mouldy Sioud, Qian Peng

**Affiliations:** 1 Department of Pathology, The Norwegian Radium Hospital, Oslo University Hospital, N-0379 Oslo, Norway; Sagar.Darvekar@rr-research.no (S.D.); Petras.Juzenas@rr-research.no (P.J.); Morten.Oksvold@rr-research.no (M.O.); Andrius.Kleinauskas@rr-research.no (A.K.); Toril.Holien@ntnu.no (T.H.); Eidi.Christensen@ntnu.no (E.C.); 2 Department of Clinical and Molecular Medicine, NTNU-Norwegian University of Science and Technology, N-7491 Trondheim, Norway; 3 Department of Hematology, St. Olavs University Hospital HF, N-7491 Trondheim, Norway; 4 Department of Dermatology, St. Olavs University Hospital HF, N-7491 Trondheim, Norway; 5 Department of Radiation Biology, Institute for Cancer Research, The Norwegian Radium Hospital, Oslo University Hospital, N-0379 Oslo, Norway; Trond.Stokke@rr-research.no; 6 Department of Cancer Immunology, Institute for Cancer Research, The Norwegian Radium Hospital, Oslo University Hospital, N-0379 Oslo, Norway; 7 Department of Optical Science and Engineering, The School of Information Science and Technology, Fudan University, Shanghai 200433, China

**Keywords:** extracorporeal photopheresis, photodynamic therapy, cutaneous T cell lymphoma, graft-versus-host disease, 5-aminolevulinic acid, 8-methoxypsoralen, protoporphyrin IX, heme biosynthesis pathway, PBMC, T-cell activation

## Abstract

Extracorporeal photopheresis (ECP), a modality that exposes isolated leukocytes to the photosensitizer 8-methoxypsoralen (8-MOP) and ultraviolet-A (UV-A) light, is used to treat conditions such as cutaneous T-cell lymphoma and graft-versus-host disease. However, the current procedure of ECP has limited selectivity and efficiency; and produces only partial response in the majority of treated patients. Additionally, the treatment is expensive and time-consuming, so the improvement for this modality is needed. In this study, we used the concept of photodynamic therapy (PDT) with 5-aminolevulinic acid (ALA), a precursor of an endogenously synthesized photosensitizer protoporphyrin IX (PpIX) in combination with blue light to explore the possibility of targeting activated human blood T cells ex vivo. With various T-cell activation protocols, a high ALA-induced PpIX production took place in activated CD3^+^, CD4^+^CD25^+^, and CD8^+^ T cell populations with their subsequent killing after blue light exposure. By contrast, resting T cells were much less damaged by the treatment. The selective and effective killing effect on the activated cells was also seen after co-cultivating activated and resting T cells. Under our clinically relevant experimental conditions, ALA-PDT killed activated T cells more selectively and efficiently than 8-MOP/UV-A. Monocyte-derived dendritic cells (DCs) were not affected by the treatment. Incubation of ALA-PDT damaged T cells with autologous DCs induced a downregulation of the co-stimulatory molecules CD80/CD86 and also upregulation of interleukin 10 (IL-10) and indoleamine 2,3-dioxygenase expression, two immunosuppressive factors that may account for the generation of tolerogenic DCs. Overall, the data support the potential use of ALA-PDT strategy for improving ECP by selective and effective killing of activated T cells and induction of immune tolerance.

## 1. Introduction

Extracorporeal photopheresis (ECP) is a cell-based immuno-modulatory treatment modality that exposes isolated leukocytes ex vivo to the photosensitizer 8-methoxypsoralen (8-MOP) followed by ultraviolet A (UV-A) radiation before infusion back to the patient in vivo. ECP was officially approved three decades ago for the treatment of cutaneous T-cell lymphoma (CTCL) [1]. Since then, this treatment has been applied for a number of T-lymphocyte (T cell) mediated diseases, including graft-versus-host disease (GvHD), organ transplant rejection, and certain autoimmune diseases [2,3,4]. However, the exact mechanism by which ECP exerts its immune modulatory effects is not fully understood. It has been suggested that cell-specific proteins released by ECP-induced apoptotic T cells are phagocytosed by dendritic cells (DCs), leading to their conversion into tolerogenic DCs that eventually suppress T cell activation [5]. These tolerogenic DCs can also induce regulatory T cells (Tregs) for immune tolerance [6,7,8].

In spite of the usefulness of ECP for certain indications, the treatment still remains underused in the clinics mainly due to the absence of prospective randomized clinical trials and also due to insufficient knowledge of its mechanisms of action. In addition, 8-MOP forms covalent DNA inter-strand crosslinking in both ailing and normal cells with no selectivity. The use of 8-MOP and UV-A (PUVA) for psoriasis is known to have an increased risk of skin carcinogenesis [9,10,11,12]. Moreover, the current form of ECP is costly, time-consuming, and results only in partial response in majority of treated patients. Hence, there is an urgent need for non-toxic, cheap, short duration, more selective and effective treatment option.

Photodynamic therapy (PDT) is a two-step procedure, typically involving the administration of a lesion/tumor-localizing photosensitizer, followed by its activation with light of a specific wavelength. PDT exploits the destructive power of reactive oxygen species (ROS) that is produced by photochemical reactions upon interaction among photosensitizer, visible light, and molecular oxygen to induce photodamage to cells [13]. PDT has several advantages over classical anticancer treatments such as surgery, ionizing radiation, and chemotherapy with non-invasive, low mutagenic potential, and low systemic toxicity [14,15], and has thus been established as a clinical treatment modality for several malignant and nonmalignant diseases [13]. However, PDT has a major side-effect of skin phototoxicity when it is carried out by using chemically synthesized photosensitizers, ultimately restricting the clinical applications. Hence, substantial interest has been aimed at developing a PDT modality that utilizes an endogenously synthesized photosensitizer [16,17]. The production and accumulation of endogenous protoporphyrin IX (PpIX) in highly proliferative cells by exogenous 5-aminolevulinic acid (ALA) are well described in the literature [16,17,18,19]. Such PpIX synthesis takes place in the mitochondria of cells due to an increased capacity of porphobilinogen deaminase and a limited activity of ferrochelatase in the heme biosynthesis pathway [16,17]. PpIX has good fluorescent and photosensitizing properties. The initial study by Malik et al. more than 30 years ago demonstrated the potential PDT application of ALA in blood malignant cells in vitro [20]. Today, topical PDT of actinic keratosis and basal cell carcinoma with ALA is widely used and Gliolan-ALA has been approved by the European Medicines Agency (EMA) and U.S. Food and Drug Administration (FDA) for fluorescence-guided surgical resection of glioma after systemic administration [21,22].

In order to explore the possibility of using ALA instead of 8-MOP for ECP, we have in the present study, evaluated the parameters affecting ALA-induced PpIX production in T cells isolated from healthy donor peripheral blood mononuclear cells (PBMCs). Furthermore, we have investigated selective and effective killing effects of ALA-PDT on activated T cells and possible mechanism of action on treated cells.

## 2. Results

### 2.1. Cell Cycle, Mitosis, Cell Proliferation, and ALA-Induced PpIX Production after T Cell Activation

**Figure 1:**
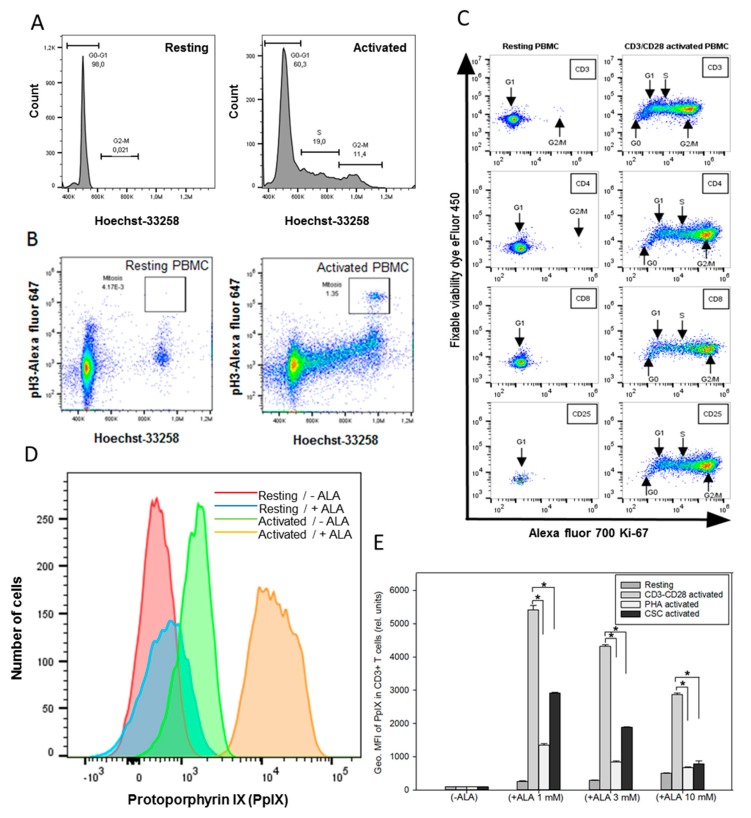
(**A** and **B**) Cell cycle and mitosis measurements. PBMCs from healthy donor were activated in vitro with anti-CD3/CD28 antibodies for three days. Resting and activated cells were fixed in methanol and stored at −20 °C until use. Cells were stained with rabbit anti-phospho-Histone H3 followed by Alexa fluor 647 donkey anti-rabbit IgG and Hoechst 33258 dye before the measurements of cell cycle and mitosis by flow cytometry. (**C**) Resting and activated cells were divided into two groups and stained for CD4/CD25/Ki-67 or CD3/CD8/Ki-67 + fixable viability dye. (**D**) ALA-induced PpIX in resting and activated CD3^+^ T cells. Histograms of PpIX fluorescence in resting cells and anti-CD3/CD28 activated cells with (24 h of ALA incubation) and without ALA were compared; (**E**) effects of T cell activation protocols on ALA-induced PpIX production. Healthy donor PBMCs were activated in vitro with anti-CD3/CD28 for three days or overnight with phytohemagglutinin (PHA) or cell stimulation cocktail (CSC), and then incubated with ALA for 24 h before the measurement of the PpIX content in CD3^+^ T cells by flow cytometry. The geometric mean fluorescence (Geo. MFI) intensity of PpIX was used to compare various groups. * *p* < 0.05.

Cell cycle, mitosis, and cell proliferation after T cell activation using anti-CD3 and CD28 antibodies were investigated by the staining of cells with Hoechst-33258 for DNA, anti-phospho-Histone H3 antibody for mitosis, and Ki-67 for cell proliferation. The DNA histograms showed an increased cell population at the S and G2 phases of cell cycle in activated PBMCs compared to resting PBMCs (Figure 1A). This was also consistent with an increase in mitotic cells in the activated PBMCs (Figure 1B). Furthermore, the CD3^+^, CD4^+^, CD8^+^, and CD25^+^ T cell subsets in the activated PBMCs demonstrated increased Ki-67 staining, indicating cell proliferation as compared to resting PBMCs (Figure 1C). This was particularly evident in the CD4^+^ and CD25^+^ T cell subsets. Interestingly, the sizes of both cells and their mitochondria were increased in the activated PBMCs, as confirmed by fluorescent microscopy with Rhodamine 123 staining (Figure A2).

Since the efficacy of ALA-PDT modality largely depends upon the cellular ability to produce PpIX during ALA incubation, the amounts of ALA-induced PpIX in resting and activated PBMCs were measured. As shown in Figure 1D, the histogram for the PpIX production shifts towards the right (higher PpIX amount) in the activated T cells incubated with ALA when compared to that of resting cells with or without ALA incubation (Figure 1D). The activated T cells without ALA also showed a small increase in PpIX (Figure 1D). This may be explained by the fact that the proliferative cells may use endogenous ALA more effectively to produce and accumulate some PpIX after being activated with anti-CD3/CD28 antibodies.

The effects of different T cell activation protocols on the ALA-induced PpIX production were also examined in PBMCs. As shown in Figure 1E, the various activation protocols led to a 5- to 60-fold increase in ALA-PpIX production in activated T cells compared to resting T cells. Activation with anti-CD3/CD28 antibodies induced significantly more PpIX (*p* < *0.05)* within the same doses of ALA than that with PHA or CSC in the CD3^+^ T cells.

Flow cytometry has a technical challenge when the broad fluorescence peak from PpIX is measured in combination with multi-staining procedures. In contrast, the CyTOF mass cytometer enables analysis of the expression of a large number of proteins simultaneously by using antibodies coupled to stable heavy metal isotopes using the Time-of-flight Inductively Coupled Plasma Mass Spectrometry (TOF ICPMS) technology. With more than 120 detection channels in the CyTOF mass cytometer, the maximum per-cell information can be obtained from a single sample without the need for compensation. To check if PpIX signals interfere with the measurements of other wavelengths during flow cytometry analyses, cells from the same PBMC sample were analyzed by both flow cytometry and CyTOF mass cytometry for comparison. The results from the CyTOF analysis were comparable to those obtained by flow cytometry for the different T cell subsets (Table A1).

### 2.2. Effects of the Parameters on ALA-Induced PpIX Production

**Figure 2:**
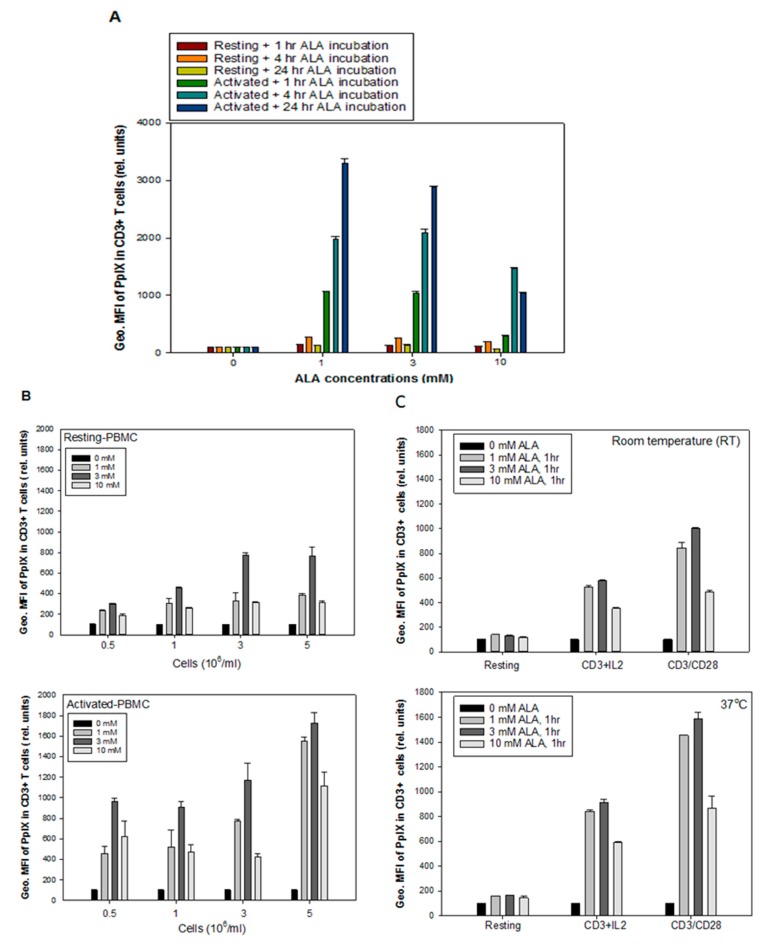
Effects of the parameters affecting ALA-induced PpIX production. Healthy donor PBMCs were activated in vitro with anti-CD3/CD28 antibodies for 3 days. (**A**) effects of different ALA incubation time intervals on PpIX production in resting and anti-CD3/CD28 activated CD3^+^ T cells; (**B**) the effect of cell density on ALA-induced PpIX production; (**C**) the effect of temperature (RT and 37 °C) on ALA-PpIX production in resting, anti-CD3/IL-2 activated and anti-CD3/CD28 activated PBMCs.

No significant cytotoxicity was observed in the resting and anti-CD3/CD28 activated CD3^+^ T cells incubated with ALA at a dose of 3 or 10 mM in the dark for 1 h (Figure A3). Figure 2A shows the effects of ALA concentrations (1, 3, and 10 mM) and incubation times (1, 4, and 24 h) on the PpIX production in the CD3^+^ T cells. Incubation with 1 mM ALA for 24 h induced the highest PpIX production. Generally, a lower ALA dose for a longer incubation time led to a higher PpIX production in the ranges of ALA concentrations and incubation times studied. However, to be clinically feasible for ALA-ECP, 1-h ALA incubation was tested in this study.

The cell density also affected the ALA-induced PpIX production. After 1 h incubation with various ALA concentrations around 3- to 5-fold increase in the PpIX level in the CD3^+^ T cells of activated PBMCs (*p* < *0.05)* as compared to the CD3^+^ T cells from resting PBMCs. An intermediate ALA dose of 3 mM at a cell density of 3 to 5 × 10^6^ cells/mL produced the highest amount of PpIX in the CD3^+^ T cells from activated PBMCs (Figure 2B).

Since the clinical standard photopheresis treatment is performed at a room temperature (20–25 °C), the effect of temperature on ALA-PpIX production was also studied. Two activation protocols of anti-CD3/CD28 and anti-CD3/IL-2 were used to compare two different temperatures (RT and 37 °C) on PpIX production using ALA at a dose of 1, 3 or 10 mM for 1 h incubation. Around 1.5-fold increase in the PpIX level was seen in the activated T cells incubated at 37 °C as compared to RT for all ALA concentrations used (Figure 2C, *p* < 0.05). ALA doses of 1 and 3 mM induced a higher amount of PpIX compared to 10 mM, independent of the incubation temperature. The activation with anti-CD3/CD28 was more efficient than anti-CD3/IL-2 in the induction of PpIX production from ALA, both at RT and 37 °C. Both temperatures did not affect the ALA-PpIX levels in the resting CD3^+^ T cells (Figure 2C).

### 2.3. ALA-PpIX-Mediated Apoptosis and Necrosis

**Figure 3:**
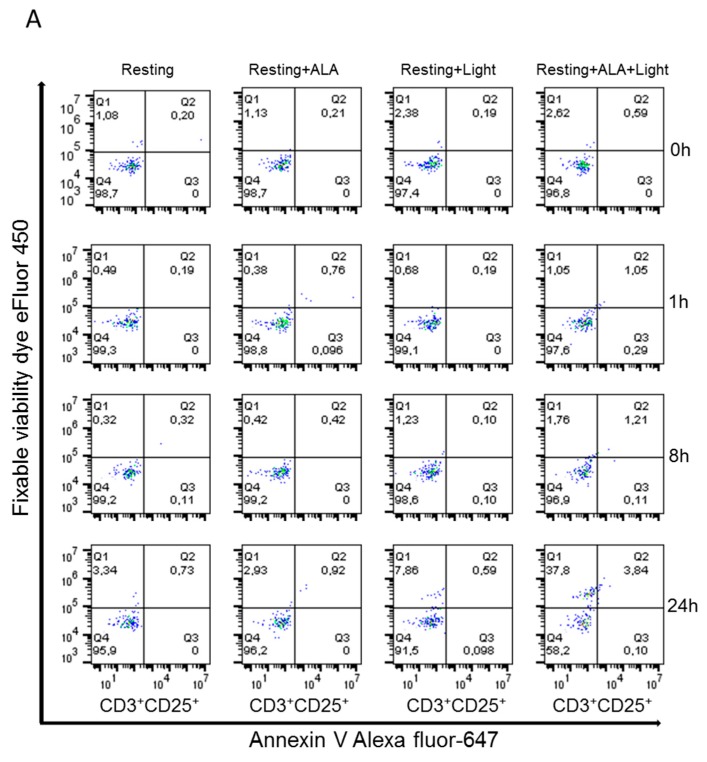

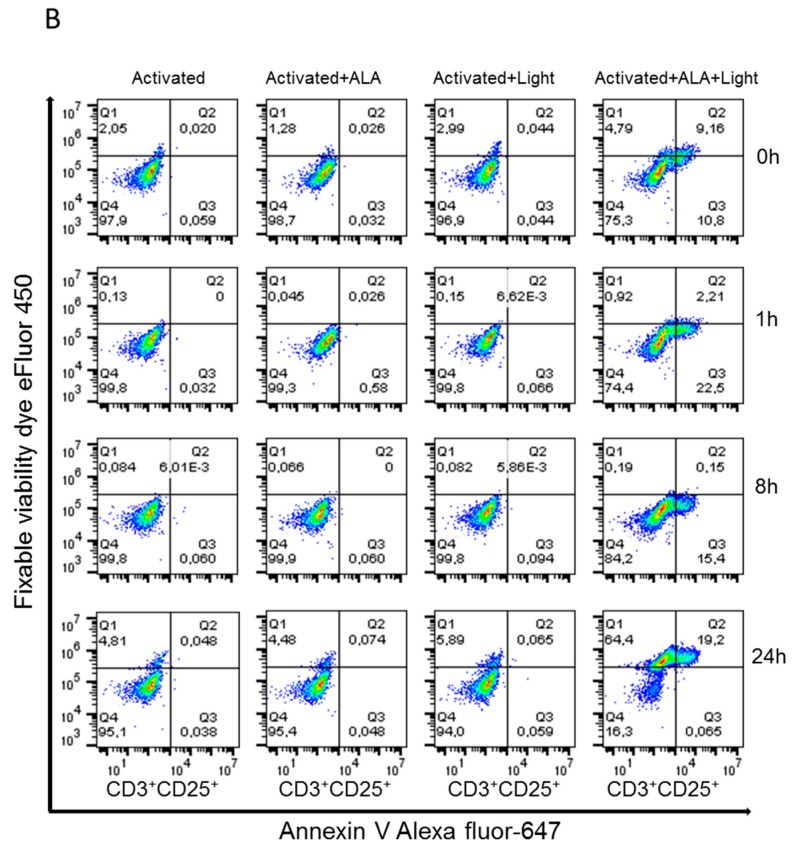
ALA-PDT mediated apoptosis and necrosis of CD3^+^CD25^+^ T cells in PBMCs. Healthy donor PBMCs were activated in vitro with anti-CD3/CD28 antibodies for three days, followed by ALA incubation (3 mM) for 1 h in the dark. The cells were then irradiated with LED blue light (30 mW/cm^2^, 9 J/cm^2^). Apoptosis and necrosis at 0, 1, 8, and 24 h after irradiation were measured by flow cytometry with annexin V/fixable viability dye staining. The cells with both negative annexin V and fixable viability dye staining were considered as viable cells. (**A**) Representative data from resting PBMCs; (**B**) Representative data from activated PBMCs.

**Figure A1:**
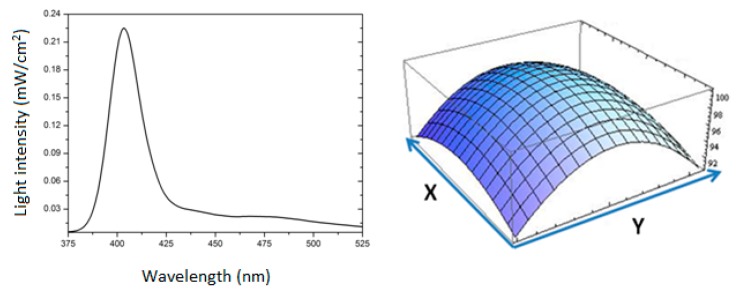
LED lamp with its emission spectrum and surface light distribution. For details refer to point 4.8 in materials and methods section.

Apoptosis and necrosis in the resting and anti-CD3/CD28 activated PBMCs subsequent to ALA-PDT treatment were evaluated. A moderate dose of ALA (3 mM for 1 h incubation) with an adequate amount of a LED blue light (30 mW/cm^2^, 9 J/cm^2^, Figure A1) was used to generate maximum amounts of apoptosis and necrosis in the cells. Cells were collected at 1, 8, and 24 h after PDT and co-stained with antibodies against CD3^+^ and CD25^+^ in combination with annexin V/fixable viability dye. In the resting PBMCs, the frequency of apoptotic and necrotic cells was low until 8 h after PDT. After 24 h, increased apoptosis and necrosis were observed in CD3^+^CD25^+^ T cells (4% and 38%, respectively; Figure 3A). In the activated cells treated with ALA and LED blue light, early apoptosis was observed in 11–23% of the cells during the period of 0–8 h after irradiation (Figure 3B). Necrosis was not detected at this early time points. In addition, 24 h after PDT, increased late apoptosis and necrosis were observed in the activated cells (19% and 64%, respectively; Figure 3B). The observed necrotic cell fraction could partially derive from the late apoptotic population.

### 2.4. Comparison between ALA/Blue Light and 8-MOP/UV-A

**Figure 4:**
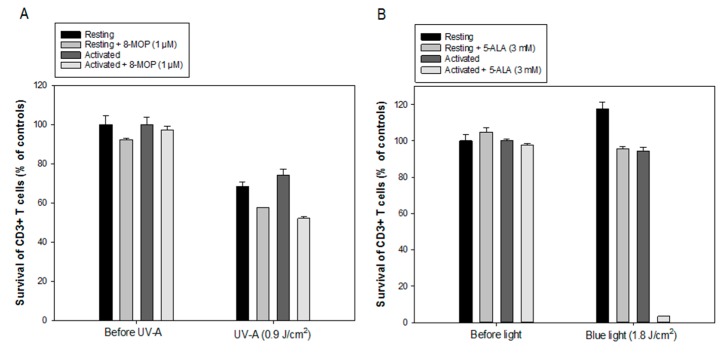
Comparison between ALA/Blue light and 8-MOP/UV-A. Healthy donor PBMCs were activated in vitro with anti-CD3/CD28 antibodies for three days. The resting and activated cells were incubated with ALA (3 mM) or 8-MOP (1 µM) for 1 h in the dark at 37 °C, and then exposed to the in-house built LED blue light or UV-A light. The CD3^+^ cell survivals were measured at 20 h after irradiation with flow cytometry using annexin-V and fixable viability dye. (**A**) Survivals of CD3^+^ T cells after 8-MOP/UV-A; (**B**) Survivals of CD3^+^ T cells after ALA-PDT.

Resting and anti-CD3/CD28 activated PBMCs were incubated with ALA (3 mM) or 8-MOP (1 µM, a concentration used for the clinical standard ECP) for 1 h in the dark, followed by irradiation with LED blue light (30 mW/cm^2^, 1.8 J/cm^2^, Figure A1) or UV-A light (0.6 mW/cm^2^, 0.9 J/cm^2^, a dose used for ECP). The viability of CD3^+^ T cells was measured 20 h after treatment. As shown in Figure 4, the percentages of dead CD3^+^ T cells were significantly higher in the ALA-PDT treated PBMCs compared to the 8-MOP/UV-A treated cells. UV-A alone killed approximately one third of the CD3^+^ resting and activated T cells (Figure 4A). 8-MOP/UV-A treatment induced some cell death in both resting and activated T cells with no selectivity, but still more than 50% of the cells survived the treatment (Figure 4A). In contrast, the LED blue light alone did not affect the survival of the resting and activated CD3^+^ T cells (Figure 4B). ALA-blue light induced a massive eradication of activated CD3^+^ T cells (95% cell death), while resting CD3^+^ T cells mostly survived (20% cell death) (Figure 4B). These data indicate that ALA-PDT is more effective and selective than 8-MOP/UV-A to eradicate CD3^+^ activated T cells, but it should be noted that these experiments aimed at comparing clinically relevant protocols of the two treatments rather than ALA and 8-MOP (in terms of their concentrations and light doses).

### 2.5. ALA-PpIX Production and PDT of Subpopulations of T Cells

**Figure 5:**
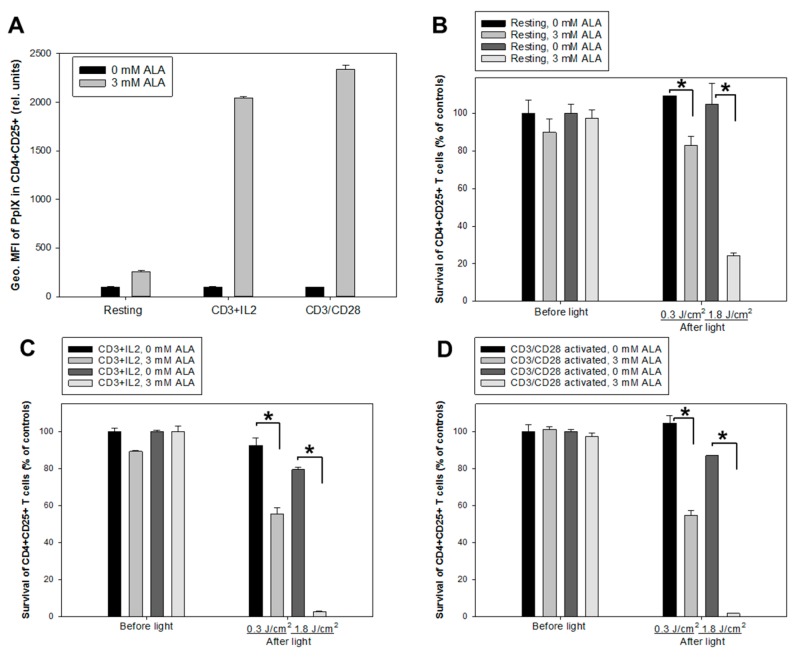
ALA-induced PpIX production and PDT of CD4^+^CD25^+^ T cells. Healthy donor PBMCs were either activated in vitro with anti-CD3/IL-2 or anti-CD3/CD28 or kept non-activated (resting) for three days. (**A**) geometric mean fluorescence intensity (Geo. MFI) of PpIX in CD4^+^CD25^+^ cells incubated with 3 mM ALA for 1 h at 37 °C after anti-CD3/IL-2 or anti-CD3/CD28 activation; (**B**, **C** and **D**) resting and activated cells were incubated with 3 mM ALA for 1 h and then exposed to the in-house built LED blue light as indicated. The survivals of CD4^+^CD25^+^ T cells were measured before light or 20 h after light exposure with flow cytometry as described in Figure 3; (**B**) for resting cells; while (**C** and **D**) for activated cells with anti-CD3/IL-2 and anti-CD3/CD28, respectively. * *p* < 0.05.

**Figure 6:**
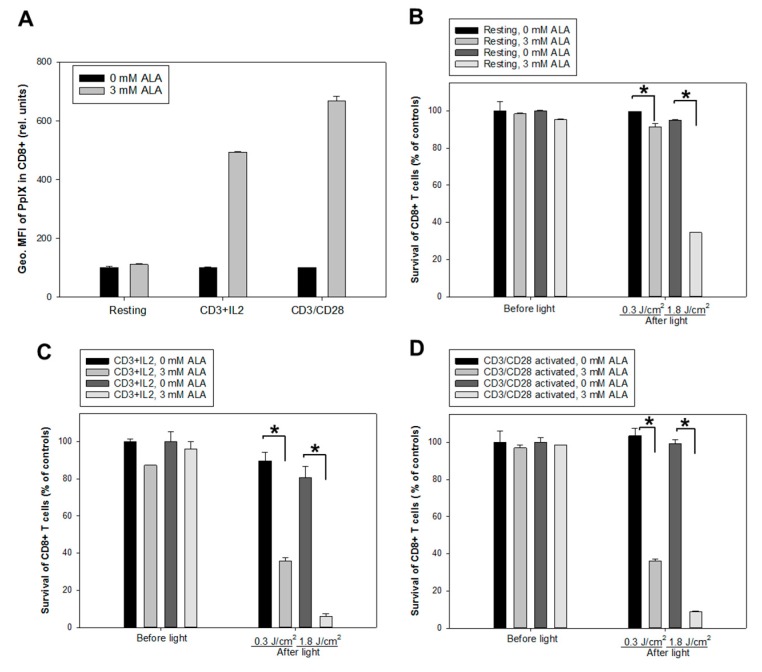
ALA-induced PpIX production and PDT of CD8^+^ T cells. Healthy donor PBMCs were either activated in vitro with anti-CD3/IL-2 or anti-CD3/CD28 or kept non-activated (resting) for 3 days. (**A**) geometric mean fluorescence intensity (Geo. MFI) of PpIX in CD8^+^ cells incubated with 3 mM ALA for 1 h at 37 °C after anti-CD3/IL-2 or anti-CD3/CD28 activation; (**B**, **C** and **D**) resting and activated cells were incubated with 3 mM ALA for 1 h and then exposed to the in-house built LED blue light as indicated. The survivals of CD8^+^ T cells were measured before light or 20 h after light exposure with flow cytometry as described in Figure 3. (**B**) for resting cells, while (**C** & **D**) for activated cells with anti-CD3/IL-2 and anti-CD3/CD28, respectively. * *p* < 0.05.

Two activation protocols using anti-CD3/IL-2 or anti-CD3/CD28 antibodies were employed to study ALA-induced PpIX production and PDT effects on activated CD4^+^CD25^+^ and CD8^+^ T cell populations. The non-activated and activated cells were incubated with ALA (3 mM) for 1 h. Figure 5A and Figure 6A) show an increased PpIX production in the activated CD4^+^CD25^+^ and CD8^+^ T cell subsets compared to the resting CD4^+^CD25^+^ and CD8^+^ cells (up to 10 and 7 times higher, respectively). Furthermore, the cells activated with anti-CD3/CD28 generated slightly but significantly more PpIX from ALA than those activated with anti-CD3/IL-2 (Figure 5A and Figure 6A).

Two doses of the LED blue light (0.3 J/cm^2^ and 1.8 J/cm^2^, Figure 1A) were used to study PDT effects on non-activated and activated cells using anti-CD3/IL-2 or anti-CD3/CD28. The survivals of the activated CD4^+^CD25^+^ and CD8^+^ T cell subsets were lower than those of non-activated cells after PDT with both light doses (Figure 5; Figure 6). PDT with a 1.8 J/cm^2^ light exposure killed almost all the activated CD4^+^CD25^+^ and CD8^+^ T cells. Interestingly, there was a PDT-mediated killing effect on the non-activated CD4^+^CD25^+^ and CD8^+^ T cells (Figure 5B and Figure 6B). This effect could result from a small amount of PpIX produced in CD25^+^ proliferative cells (Figure 5A) and CD8^+^ T cells (Figure 6A). The activation protocols did not affect the cell survivals with or without ALA before light irradiation (Figure 5C,D and Figure 6C,D).

### 2.6. ALA-PDT of Mixed Populations of Resting and Activated T Cells

**Figure 7:**
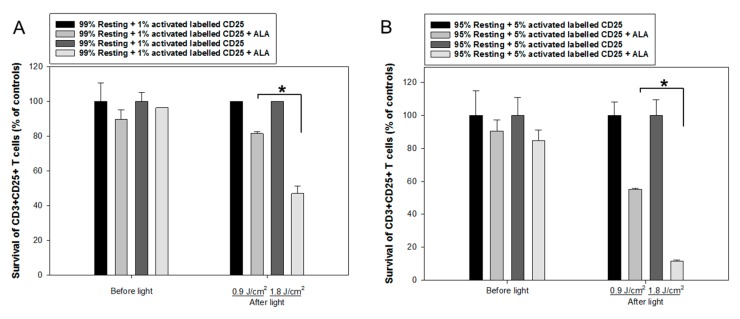
ALA-PDT of mixed populations of resting and activated cells. Healthy donor PBMCs were activated in vitro with anti-CD3/CD28 antibodies for three days. The activated T cells were then labeled with anti-human CD25-FITC antibody. The resting and CD25-FITC labeled activated T cells were mixed in certain ratios as indicated. The mixed cells were incubated with 3 mM ALA for 1 h at 37 °C and then irradiated with the LED blue light at 0.9 J/cm^2^ or 1.8 J/cm^2^. The cell survivals were measured 2 h after light irradiation with flow cytometry as described in Figure 3. The control samples without light are also included. (**A**) Mixture of 1% CD25-FITC labelled activated T cells with 99% resting PBMCs. (**B**) Mixture of 5% CD25-FITC labelled activated T cells with 95% resting PBMCs. * *p* < 0.05.

Generally, the anti-CD3/CD28 activation causes >99% resting CD3^+^/CD25^-^ T cells to be activated CD3^+^/CD25^+^ T cells. To further study the selectivity of ALA-PDT of activated CD3^+^/CD25^+^ T cells, a co-culture of non-activated and anti-CD3/CD28 activated PBMCs was performed. The activated T cells were initially labeled with anti-CD25-FITC after being activated. Since our unpublished data have shown only 1 to 5% CD3^+^/CD25^+^ diseased T cells in the blood of standard ECP-treated patients with graft versus host disease, the co-cultures were made in the following ratios: 99% resting PBMCs + 1% CD25-FITC labeled activated T cells or 95% resting PBMCs + 5% CD25-FITC labeled activated T cells. The co-cultures were incubated with 3 mM ALA for 1 h and then irradiated with LED blue light at 0.9 J/cm^2^ or 1.8 J/cm^2^ (30 mW/cm^2^, Figure 1A). The survival of the labeled CD25^+^ T cells was measured by flow cytometry of the CD25-FITC signals together with anti-CD3, annexin V and fixable viability dye. Such experimental design made possible to only target those activated CD3^+^ T cells with previously anti-CD25-FITC labeled, although an anti-CD25 antibody was not used for such measurement. In the co-culture with 1% activated CD25^+^ T cells PDT with 0.9 J/cm^2^ or 1.8 J/cm^2^ light exposure induced 20% and 50% of cell death of the activated CD25^+^ T cells, respectively (Figure 7A). This PDT-induced cell death was increased to 50% and 90% in the co-culture containing 5% CD25^+^ activated T cells (Figure 7B). With increased light doses and/or ALA incubation duration, a higher proportion of activated T cells is expected to be eradicated. These results clearly demonstrate that ALA-PDT enables to selectively kill the activated T cells in the mixture of resting and activated T cells.

### 2.7. Monocyte-Derived DCs Are Resistant to ALA-PDT

Enriched populations of PBMC were used to grow monocyte-derived DCs without exogenous growth factors and cytokines added. DCs were adhered to the bottom of a flask after 8–10 days and were easily distinguished from normal PBMCs due to their larger size and attachment. The adhered DCs were detached by gentle scraping and then incubated with various concentrations of ALA (1, 3 & 10 mM) for 1 h before being exposed to the LED blue light at 1.8 J/cm^2^(Figure 1A). The treated DCs were further incubated for 24 h at 37 °C before the cell survival was measured with the CTG assay. The human Jurkat T cell lymphoma cells were included as a positive control.

**Figure 8:**
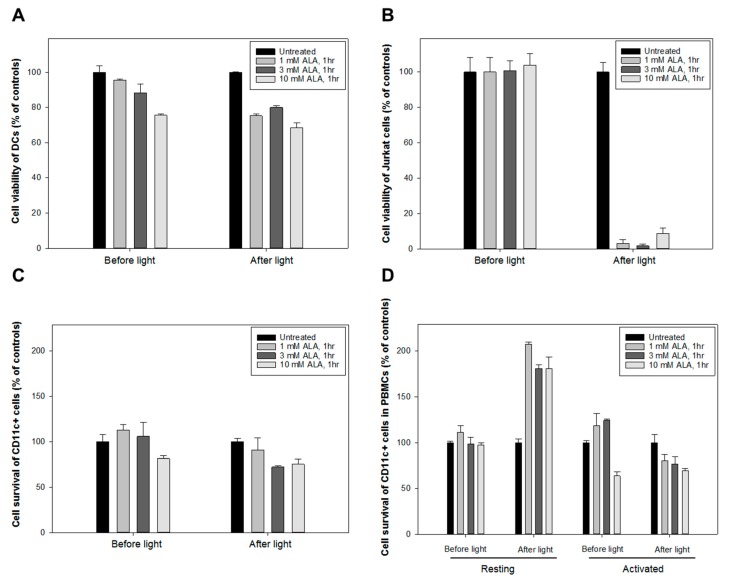
Monocyte-derived dendritic cells (DCs) resistant towards ALA-PDT. CellTiter Glo^®^ (CTG) luminescent cell viability assay was performed on DCs and Jurkat cells. Up to 3 × 10^5^ DCs or Jurkat cells/well were seeded in the Corning^®^ 96-well white polystyrene microplates and incubated at 37 °C for 24 h. The cells were then incubated with ALA at various concentrations for 1 h and irradiated with the LED blue light at 1.8 J/cm^2^. The cell survivals were measured at 24 h after light exposure. (**A**) for DCs, (**B**) for Jurkat cells, (**C**) for pure CD11c^+^ DCs, and (**D**) for CD11c^+^ DCs isolated from anti-CD3/CD28 activated PBMCs.

Figure 8A shows that DCs were relatively resistant to the ALA-PDT treatment (60–70% survival at all doses of ALA). In contrast, the Jurkat cells were highly sensitive to the same treatment and only 3–10% survived (Figure 8B).

In another experiment. purified CD11c^+^ DCs sorted from PBMCs by flow cytometry (Figure 8C) were compared with the CD11c^+^ subpopulation in the PBMCs (Figure 8D) with or without anti-CD3/CD28 activation. In both cases, the CD11c^+^ DCs showed to be relatively resistant to the killing effect of ALA-PDT (Figure 8C,D).

### 2.8. ALA-PDT-Treated T Cells Induce Tolerogenic DCs

**Figure 9:**
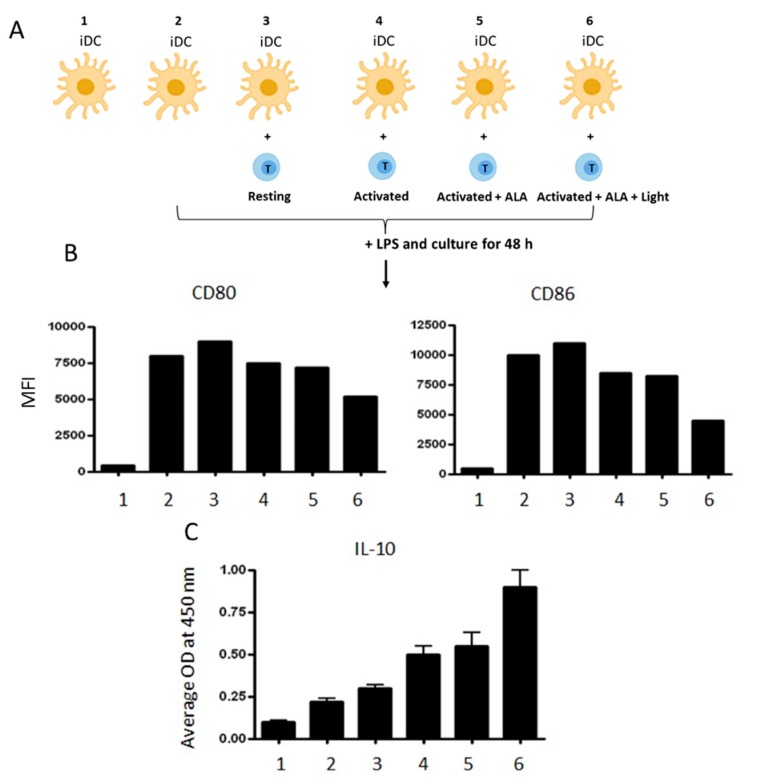
ALA-PDT treated T cells induce tolerogenic DCs. (**A**) monocyte-derived immature DCs (iDC) were co-cultured with autologous ALA-PDT damaged CD4^+^ T cells and then stimulated with LPS for 48 h as illustrated; (**B**) a representative example of expression of the co-stimulatory molecules CD80 and CD86 analyzed by flow cytometry; (**C**) IL-10 content in the culture supernatants measured by ELISA. The data is a representative of three independent experiments.

Immunosuppressive effects of ALA-PDT-damaged CD4^+^ T cells on autologous DCs were investigated. The purified autologous CD4^+^ T cells were activated with anti-CD3/CD28 antibodies before ALA-PDT treatment. The PDT-induced apoptotic/necrotic CD4^+^ cells were then co-cultured with autologous immature DCs (iDC), followed by a further activation with LPS for 48 h as illustrated in Figure 9A. Downregulation of the co-stimulatory molecules CD80 and CD86 was observed in the DCs co-cultured with the activated CD4^+^ T cells previously treated with ALA-PDT. The observed downregulation of CD80 and CD86 expression was less prominent in the DCs co-cultured with activated T cells treated with ALA alone (Figure 9B). This suggests that apoptotic/necrotic T cells have suppressed LPS-induced maturation of iDCs. Additionally, ALA-PDT-treated T cells promoted the release of IL-10 from DCs when compared to DCs co-cultured with control T cells (Figure 9C). IL-10 is an immunosuppressive cytokine that inhibits T cell function and favors the conversion of naïve T cells into regulatory T cells.

### 2.9. ALA-PDT-Treated T Cells Induce the Expression of Indoleamine 2, 3-Dioxygenase

**Figure 10:**
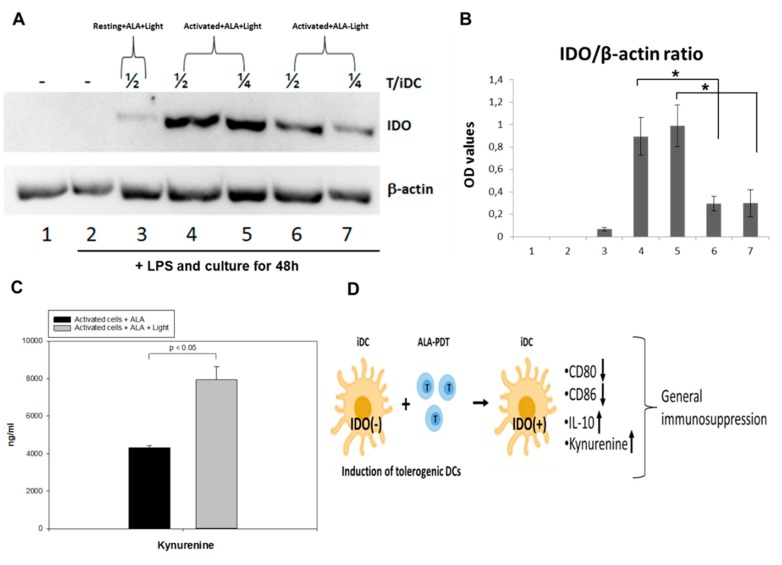
ALA-PDT treated T cells induce expression of IDO. (**A**) monocyte-derived immature DCs were incubated with control or PDT-treated autologous CD4^+^ T cells at a ratio of 1/2 (4 & 6) or 1/4 (5 & 7) (as indicated) overnight and then stimulated with LPS (50 ng/mL) for further 48 h. The IDO expression was analyzed by Western immunoblotting. Data is a representative of three independent experiments; (**B**) normalized OD values of IDO expression with β-actin; (**C**) the Kynurenine contents in the supernatants of activated cells treated with ALA alone or plus light were measured with ELISA; (**D**) a simple schematic illustration of the suggested mechanism on how ALA-PDT mediated over-expressed IDO in iDCs induces an immune-suppressive effect with the downregulation of CD80 and CD86 and increased expression of IL-10 and Kynurenine.

Indoleamine 2, 3-dioxygenase (IDO) is a rate-limiting enzyme for the tryptophan catabolism that plays an important role in the induction of immune tolerance [23,24]. Tryptophan is an essential amino acid required for T cell proliferation and survival. Tolerogenic DCs are known to express IDO in responses to various stimuli [25,26]. The expression of IDO in DCs co-cultured with autologous CD4^+^ T cells (with a ratio of 1/2 or 1/4) treated with or without ALA-PDT was measured by Western blotting. DCs incubated with activated CD4^+^ T cells previously treated with ALA-PDT significantly upregulated IDO expression when compared to the treatment with ALA alone, although a slight upregulation of IDO was also noticed in the control DCs incubated with activated CD4^+^ cells pretreated with ALA alone (Figure 10A,B and Figure S1). DCs incubated with the resting CD4^+^ T cells treated with ALA-PDT did not show any IDO over-expression (Figure 10A,B and Figure S1). Furthermore, the level of kynurenine, a specific catabolic product of tryptophan, was found to be higher in the PDT treated samples than in the control with ALA alone (Figure 10C). These data suggest that the interaction, phagocytosis, and processing of ALA-PDT-mediated apoptotic and necrotic CD4^+^ T cells by iDCs may induce the conversion of immunogenic DCs to tolerogenic DCs with the main function of suppressing immune responses (Figure 10D).

## 3. Discussion

In the present study, we explored the possibility of using ALA and blue light to selectively induce photodamage to activated T cells ex vivo. This modality was tested in T cells from PBMCs isolated from healthy human donors in order to better understand possible mechanisms of action of ALA-PDT in our ongoing clinical ECP trial using ALA in patients with CTCL or chronic GvHD (https://clinicaltrials.gov/ct2/show/NCT03109353).

**Figure A2:**
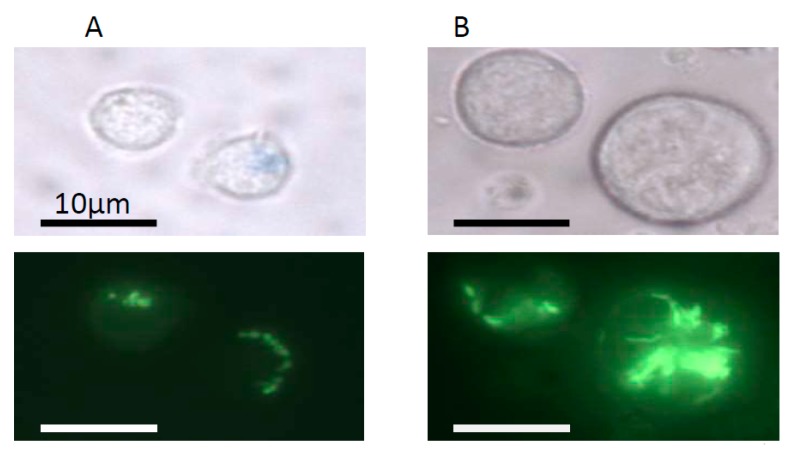
Sizes of cells and mitochondria in resting (**A**) and activated PBMCs (**B**). Resting and activated PBMCs without ALA treatment were seeded on MatTek glass bottom Petri dishes. Mitochondria were stained with 1 μM Rhodamine 123 (Rh123). For imaging, a Zeiss Axiovert 40CFL microscope with a 100× objective (1.25 NA) was used. For Rh123 detection, the filter combination was composed of a 450–490 nm (peak at 475–485 nm) excitation BP filter and a >515 nm emission LP filter.

**Figure A3:**
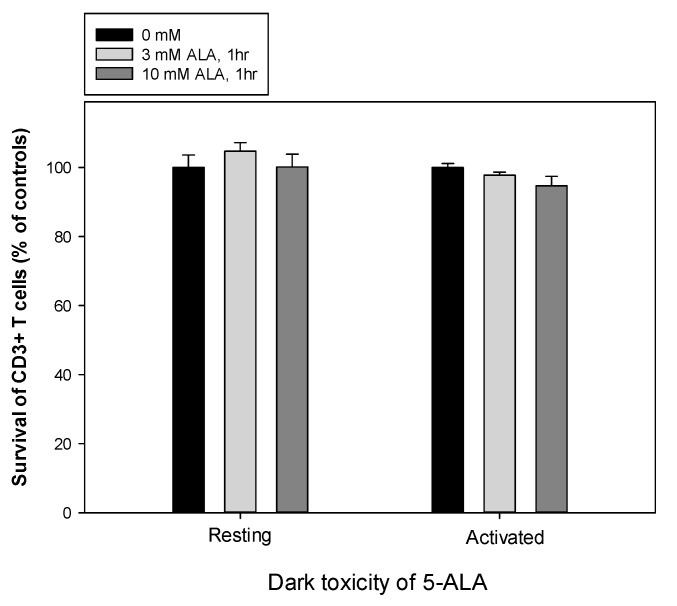
Dark toxicity of ALA on resting and activated CD3^+^ T cells. Healthy donor PBMCs were activated in vitro with anti-CD3/CD28 antibodies for three days. The resting and activated PBMCs were incubated with 0, 3, and 10 mM ALA for 1 h in the dark. The cells labeled with anti-CD3, fixable viability dye, and annexin V were analyzed by flow cytometry as described in Figure 3.

All activation protocols tested in the present study induced a high amount of PpIX from ALA in the CD3^+^, CD4^+^, and CD8^+^ T cell subsets. However, T cells activated with the anti-CD3/CD28 antibodies activation protocol produced a significantly higher amount of ALA-PpIX than those activated with the PHA, CSC, or CD3/IL-2 activation protocols (Figure 1E, Figure 2A–C, Figure 5A and Figure 6A). Notably, anti-CD3/CD28 antibodies provided primary and co-stimulatory signals to activate T cells (without the need of feeder cells) and increased the cellular and mitochondrial size and granularity (Figure A2). This may contribute to the enhanced ALA-induced PpIX production.

Chelation of iron into PpIX is the last step before heme synthesis and a low intracellular iron level may thus cause a high PpIX production from ALA. Generally, proliferative and activated cells have a relatively low iron level probably due to high consumption of iron, although they often have a high expression of transferrin receptor (CD71) that is needed for iron uptake. In the present study, the activated CD3^+^ T cells with a high CD71 expression had a higher ALA-derived PpIX production than the resting CD3^+^ T cells with a low CD71 expression. Similar results were also obtained by others [19,27].

The production of PpIX from ALA is determined by a number of factors, including ALA dose, ALA incubation time, temperature, and cell density. The higher PpIX production observed after ALA incubation for 4 or 24 h at the dose of 1 mM or 3 mM compared to 10 mM (Figure 2A) suggests that high amounts of ALA, to some extent, affect the activities of enzymes involved in the heme biosynthesis pathway, although the cell survival is not affected. Furthermore, a cell density of 5 × 10^6^/mL produced more ALA-PpIX as compared to lower cell densities at all ALA doses studied (Figure 2B), indicating that the 5 × 10^6^/mL cell density might favor cells to synthesize PpIX from ALA. Cell densities higher than 5 × 10^6^/mL with a short 1 h incubation may require more ALA to produce a therapeutic amount of PpIX. The temperature during ALA incubation is another factor affecting PpIX production. The activated T cells incubated with ALA at 37 °C produced a significantly higher amount of ALA-PpIX than at a room temperature (Figure 2C) probably due to the fact that 37 °C is optimal for the enzyme activities in the heme biosynthesis pathway. It should be noted that a therapeutic amount of PpIX can still be produced at RT for 1 h at the concentrations of 3 or 10 mM ALA. The ALA-induced PpIX was primarily confined to the mitochondria of the T cells, in agreement with our previous finding using human Reh leukemia cell line incubated with ALA hexylester [28]. The enlarged mitochondria in the activated T cells observed in this study may promote ALA-PpIX production. It should be pointed out that the parameters affecting ALA-induced PpIX in activated human PBMCs need to be confirmed in patient’s leukocyte-rich buffy coat or mononuclear cells collected from the commercial Therakos Photopheresis System or apheresis devices.

No significant cytotoxic effects of ALA alone were seen in this study with doses up to 10 mM, a consistent result with our previous finding using the CD45^+^ leukocytes from six 8-MOP-ECP-treated patients [18]. Generally, the killing effect of ALA-PDT depends on the intracellular amount of PpIX produced as well as the light dose used. ALA-PDT with LED blue light exposure at 0.3 J/cm^2^ and 1.8 J/cm^2^ (30 mW/cm^2^) in this study effectively killed CD4^+^CD25^+^ and CD8^+^ T cells from activated PBMCs (Figure 5 and Figure 6). However, ALA-PDT with 1.8 J/cm^2^ light also, to some extent, damaged resting CD4^+^CD25^+^ and CD8^+^ T cells from non-activated PBMCs (Figure 5 and Figure 6). In order to better understand the selective killing effect on activated T cells rather than resting T cells, co-culture experiments were performed and activated CD25^+^ T cells were more selectively killed by ALA-PDT. (Figure 7A,B).

As compared with the reduced selective killing effect of activated CD3^+^ T cells with 8- MOP/UV-A at a clinically applied dose (Figure 4A), ALA-PDT treatment caused a more selective eradication of activated CD3^+^ T cells (Figure 4B). This finding is consistent with our previous report comparing the effects between 8-MOP/UV-A and ALA/UV-A on T cells obtained from four cGvHD patients [18]. This may provide a significant clinical impact on targeting proliferative diseased T cells with ALA-PDT.

Although the mechanisms responsible for clinical benefits of 8-MOP-ECP remain elusive, it is generally assumed that the combination of 8-MOP with UV-A induces apoptosis of T cells ex vivo followed by phagocytosis of the apoptotic cells by DCs to induce immune tolerance [29]. In the present study, both apoptosis and necrosis of activated T cells were selectively and effectively induced by ALA-PDT (Figure 3), while DCs were resistant to the treatment (Figure 8). Furthermore, the decreased expression of co-stimulatory molecules of CD80 and CD86 with the increased level of IL-10 (Figure 9) indicates the induction of tolerogenic DCs [30]. A similar report by others has also shown an increased expression of IL-10 in monocyte-derived DCs in the presence of apoptotic lymphocytes induced by ECP treatment with 8-MOP/UV-A [31]. The exact mechanisms that mediate immune tolerance are not fully understood, but the breakdown of tryptophan into its downstream kynurenine metabolites by the rate-limiting catabolic enzyme IDO is involved [32]. Since kynurenines are known to activate Treg cells to amplify the immuno-suppressive process, the DCs with upregulation of IDO synthesis may inhibit innate and adaptive immunity to promote immune tolerance. In this study, ALA-PDT-mediated over-expression of IDO in DCs is novel and this finding concomitantly with the increased level of kynurenines (Figure 10) may indicate an induction of an immune suppressive effect.

## 4. Materials and Methods

### 4.1. Cell Culture and Reagents

Human peripheral blood was from anonymous healthy donors at the Blood Bank (Oslo University Hospital, Norway). The human peripheral blood mononuclear cells (PBMC) were grown in RPMI-1640 growth medium (Gibco, Grand Island, NY, USA) supplemented with 10% fetal bovine serum (FBS), 100 units/mL penicillin, 100 µg/mL streptomycin, and 2 mM L-glutamine. A human T cell lymphoma cell line (Jurkat) was also cultured in the same medium. 5-Aminolevulinic acid (ALA) (Sigma Aldrich, St Louis, MO, USA) was dissolved in PBS to a concentration of 1 M and further diluted. The stock solution of 1 M 8-methoxypsoralen (8-MOP) (Sigma Aldrich) was prepared in chloroform and further diluted. The stock solution of 0.1 mg/mL phytohemagglutinin (PHA) was prepared in PBS. All the stocks mentioned above were stored at −20 °C until further use.

### 4.2. Isolation of PBMCs

Isolation of human PBMCs from different healthy donor buffy coats were carried out by using the Lymphoprep density gradient solution (Axis-Shield, Oslo, Norway). Buffy coats were layered over 5 mL of Lymphoprep density gradient solution in the 15-mL falcon tubes at room temperature (RT). The tubes were then centrifuged at 800×*g* for 30 min. The mid layer was transferred to a new falcon tube and washed twice in the growth medium containing 1% FCS by centrifugation at 100× *g* for 10 min before exclusion of excess platelets. Cells were then re-suspended in the growth medium, counted, frozen down, or freshly used in downstream experiments. Monocytes were isolated from PBMCs using plastic adherence. Monocyte-derived immature dendritic cells (iDCs) were generated by culturing adherent monocytes in the growth medium supplemented with granulocyte-macrophage colony-stimulating factor (GM-CSF) (25 ng/mL, R&D, Minneapolis, MN, USA) and interleukin (IL)-4 (50 ng/mL, R&D) for 4 or 5 days.

### 4.3. Activation of T-Cells In Vitro

CD3 antibody (eBioscience, cat.no. 16-0037-85) was diluted 1:200 in sterile PBS and used to coat T75 flasks or wells in 12- or 24-well plates for 1 h at 37 °C. The antibody solution was then removed and the flasks or wells were washed three times with sterile 1× PBS. PBMCs were diluted in the growth medium to 10 to 20 × 10^6^ cells/flask with 20 mL of the final volume or at a cell density of 2 × 10^5^ to 1 × 10^6^ cells/mL in 12- or 24-well plates. CD28 antibody (eBioscience, cat.no. 16-0289-85) at a concentration of 1 µg/mL was added to the flask or wells. The flasks or wells were further incubated for 3 days before running experiments. This protocol was also used in the experiments with pure CD4^+^ T cells (>99%) isolated from PBMC with CD4 Dynabeads® in combination with Detachabead^®^reagent (Dynal, Oslo, Norway). For comparison, cells were also activated overnight with PHA (5 µg/mL) or cell stimulation cocktail (CSC, 1×), a mixture of phorbol 12-myristate 13-acetate (PMA) and ionomycin (eBioscience, cat.no. 00-4970-93). In some experiments, expansion of a T-cell population was stimulated by using recombinant IL-2 [33]. After coating flasks/wells with anti-CD3 antibody, recombinant IL-2-human (10 U/mL) (Sigma-Aldrich, cat.no. 57600-1VL) was used to activate the T cells. The flasks or wells were further incubated for 3 days before experiments.

### 4.4. Fluorescence Microscopy

Resting and activated PBMCs without ALA were seeded on glass bottom Petri dishes (MatTek Corporation, Ashland, MA, USA). Rhodamine 123 (Rh123) (1 µM) was used for mitochondria staining. A Zeiss Axiovert 40CFL microscope (City, Country) with a 100×/1.25 objective was used to image Rh123 using the filter combination of an excitation BP filter 450–490 nm (peak at 475–485 nm) and an emission LP filter >515 nm.

### 4.5. Flow Cytometry Analyses

The samples for flow cytometry measurements were prepared as described in [18]. The following antibodies were used: CD3 PE-Cyanine 7 (eBioscience, cat.no. 25-0037-42), CD3 PE (eBioscience, cat.no. 12-0038-42), CD4 FITC (eBioscience, cat.no. 11-0049-42), CD4 PE (eBioscience, cat.no. 12-0048-42), CD4 PE-Cyanine 7 (eBioscience, cat.no. 25-0048-42), CD8a PE (eBioscience, cat.no. 12-0088-42), CD8a FITC (eBioscience, cat.no. 11-0088-42), CD25 PE (ImmunoTools, cat.no. 21270254), CD25 FITC (ImmunoTools, cat.no. 21270253), CD25 FITC (Life Technologies, cat.no. MHCD2501), Annexin V Alexa Fluor 647 (Life Technologies, cat.no. A23204), Annexin V FITC (ImmunoTools, cat.no. 31490013), fixable viability dye eFluor 450 (eBioscience, cat.no. 65-0863-18), Ki-67 Alexa Fluor 700 (BD pharmingen, cat.no 561277), rabbit anti-phospho-Histone H3 (Ser10) (Millipore, cat.no. 06-570), Alexa Fluor 647 donkey anti-rabbit (Life Technologies, cat.no. A31573).

Flow cytometry was performed to analyze the expression of co-stimulatory molecules using antibodies against CD80 and CD86 (Dako, Glostrup, Denmark). For other flow cytometry experiments, cells were fixed in methanol and stored at −20 °C until use. Cells were stained with rabbit anti-phospho-Histone H3 primary antibody on ice for 30 min followed by staining with Alexa Fluor 647 donkey anti-rabbit secondary antibody and Hoechst 33258 dye before cell cycle and mitosis measurements.

All flow cytometry measurements were performed using a Cytoflex S cytometer (Beckman Coulter Life Sciences, Indianapolis, IN, USA) with the Cytexpert 2.1 software (Beckman Coulter); and the analyses were performed using the FlowJo V10 software (Treestar, Ashland, OR, USA).

### 4.6. CyTOF

PBMCs were treated as described for flow cytometry. Resting and anti-CD3/CD28 activated cells without LED blue light irradiation were stained with cisplatin live/dead stain (5 µM in PBS) at RT for 5 min following surface staining with a panel of antibodies diluted in PBS with 1% BSA at 4 °C for 30 min. The following antibodies were used: CD45-156Gd (#3156010B), CD3-154Sm (#3154003B), CD4-145Nd (#3145001B), CD8-168Er (#3168002B), CD25-149Sm (#3143010B), all from Fluidigm (San Francisco, CA, USA). Cells were fixed in PFA (1.6%) and permeabilized in methanol before being stained with cationic nucleic acid intercalator-Ir (125 nM; Fluidigm) for 20 min at RT. The cells were then washed in PBS and resuspended in ddH_2_O and collected on a CyTOF 2.0. The mass cytometry data were evaluated using the manual gating in FlowJo and transformed with an arcsinh scale (cofactors of 15).

### 4.7. Intracellular ALA-Induced PpIX

PBMCs from healthy donors were diluted (2 × 10^5^ to 1 × 10^6^, resting or activated) and seeded per well in 12- or 24-well plates. The cells were incubated with ALA at various concentrations (1, 3 and 10 mM) for 1, 4 or 24 h in the dark at RT or 37 °C. Intracellular ALA-induced PpIX was measured with flow cytometry using a 405 nm violet laser for the excitation and a 660/20 BP emission filter for detection. The intracellular fluorescence derived from ALA-induced PpIX was quantified by the geometric mean fluorescence intensity (Geo. MFI).

### 4.8. Light Sources

The blue light source was an in-house built lamp consisting of 12 light-emitting diodes (LED) designed as a 3 × 4 array to cover the whole size of one cell culture plate. The Xeon 3 Power Violet LEDs were obtained from the OptoSupply Ltd. (Hong Kong, China). The emission spectral peak is at 405 nm with a half intensity beam angle of 70^o^. A constant 600 mA current driver (model LDD-600LW; Mean Well Enterprises Co., City, Taiwan) was used to feed the LED array (Figure A1). The lamp has a fluence rate of 30 mW/cm^2^ with an even surface light distribution (8% variation). In some experiments, a home-made UV-A lamp (Philips Th 20W/09) emitting light mainly in the region of 340–410 nm was used [34]. This UV-A lamp bears an exact resemblance of emission spectral wavelengths to those of the built-in certified UV-A source in the commercial Therakos^TM^ Photopheresis System and has a fluence rate of 0.6 mW/cm^2^.

### 4.9. PDT Treatment Ex Vivo

PBMCs were diluted and seeded in an appropriate cell density in 12- or 24-well plates; and incubated with ALA or 8-MOP for 1, 4, or 24 h in the dark. The UV-A light or the LED 405 nm light was then used to irradiate the cells. The horizontal positioning of both light sources allowed placing the cell culture plate at the center of the light sources, resembling the illumination of a patient’s buffy coat during a clinical standard ECP procedure. After irradiation, cells were incubated for 20 h at 37 °C before the analyses of cell survivals by flow cytometry.

### 4.10. CellTiter Glo^®^ (CTG) Luminescent Cell Viability Assay

The CellTiter Glo^®^ (CTG) luminescent cell viability assay (Promega, Madison, WI, USA) was performed according to the manufacturer’s instructions to determine cell viability in dendritic cells and the Jurkat human lymphoma cell line. This assay is a homogenous method to quantify the amount of ATP, an indicator of metabolically active living cells. The luminescence was measured with a Fluoroskan Ascent FL microplate fluorometer and luminometer (ThermoFisher Scientific, Waltham, MA, USA).

### 4.11. Enzyme-Linked Immunosorbent Assay (ELISA)

To study the effects of autologous PDT-damaged T cells on DC functions, resting and activated CD4^+^ T cells (2 × 10^6^ cells/mL) treated with or without ALA (3 mM, 1 h incubation) and the LED blue light (30 mW/cm^2^, 1.8 J/cm^2^) were incubated with iDCs, followed by the stimulation with lipopolysaccharides (LPS) (50 ng/mL) for 48 h. The culture supernatants were then collected to measure the IL-10 content by ELISA (PromoKine, Heidelberg, Germany) according to the manufacturer’s instructions. Kynurenine in the culture supernatants were determined by ELISA (Immusmol, Bordeaux, France) after incubation for 96 h. The expression of co-stimulatory molecules CD80 and CD86 in DCs were measured by flow cytometry. As controls, unstimulated autologous monocytes were added to T cells as non-suppressive accessory cells. The T-cell responses were analyzed by flow cytometry after 3 days of culture.

### 4.12. Western Blots

Equal amounts of protein extracts (40 µg/sample) were analyzed by polyacrylamide gel electrophoresis (PAGE) and then transferred to nitrocellulose membrane. Subsequent to blocking with 5% dry milk in PBS/Tween (0.1%) for 2 h at RT, the membranes were probed with the antibody specific to indoleamine 2,3-dioxygenase (IDO), followed by the horseradish peroxidase (HRP)-conjugated rabbit anti-human IgG antibody. After incubation with the ECL detection reagent, the signals were visualized using a ChemiDoc imaging system (BioRad,Hercules, CA, USA) Blots were also stripped and re-probed with the anti-β-actin. Finally, the blot signals were quantified using the ChemiDocXRS digital imaging system.

### 4.13. Statistical Analysis

All experiments were performed thrice in duplicates and all the figures are from representative experiments. The values were plotted as mean ± standard deviation and were compared using the Student’s *t*-test. *p*-values < 0.05 (*) were considered significant.

## 5. Conclusions

Among several factors, PpIX production in activated T cells largely depends upon ALA dose and its incubation time. The activated T cells (using anti-CD3/CD28 antibodies) induced a 5-60-fold increase in the PpIX production in the ALA-treated PBMCs as compared to resting T cells. No dark cytotoxicity of ALA was observed in T cells exposed to the concentrations up to 10 mM for 1 h. The increased production of ALA-PpIX led to apoptosis and necrosis of the activated T cells after LED blue light exposure. This killing effect was selective and effective after co-cultivating activated and resting T cells. ALA-PDT killed activated T cells more selectively and efficiently than 8-MOP/UV-A. Unlike activated T cells, DCs were resistant to ALA-PDT. IDO overexpressing tolerogenic DCs were induced after being co-cultured with ALA-PDT-damaged T cells. The findings in this study indicate that ALA-PDT may have the potential for improving the ECP selectivity and efficacy.

## Supplementary Materials

The following are available online at https://www.mdpi.com/2072-6694/12/2/377/s1, Figure S1: Monocyte-derived immature DCs were incubated with control or PDT-treated autologous CD4^+^ T cells at a ratio of 1/2 (4 & 6) or 1/4 (5 & 7) (as indicated) overnight and then stimulated with LPS (50 ng/mL) for further 48 h. The IDO expression was analyzed by Western immunoblotting. Data is a representative of three independent experiments.

## Appendix A

**Table A1:** Comparison between the data obtained from CyTOF and Flow cytometry.

Comparison between Cytof and Flow Data			
Cell types	Marks	Cytof	Flow
Resting	CD3	82.3%	88.8%
Resting + ALA		84.6%	88.4%
Activated		94.3%	94.7%
Activated + ALA		94.2%	96.3%
Resting	CD4	54.0%	57.1%
Resting + ALA		56.6%	57.6%
Activated		64.8%	58.7%
Activated + ALA		65.2%	58.6%
Resting	CD8	47.5%	31.0%
Resting + ALA		46.8%	30.6%
Activated		41.1%	33.8%
Activated + ALA		40.8%	33.8%
Resting	CD25	2.18%	3.00%
Resting + ALA		2.64%	3.08%
Activated		97.3%	96.9%
Activated + ALA		97.9%	97.2%
